# Presentations, Diagnosis, and Treatment of Post-COVID Viral Myocarditis in the Inpatient Setting: A Narrative Review

**DOI:** 10.7759/cureus.39338

**Published:** 2023-05-22

**Authors:** Michael N Sayegh, Allie E Goins, Mary Ann Kirkconnell Hall, Yoo Mee Shin

**Affiliations:** 1 Department of Medicine, Emory University School of Medicine, Atlanta, USA; 2 Department of Medicine, Division of Hospital Medicine, Emory University School of Medicine, Atlanta, USA; 3 Hospital Medicine, Emory University Hospital Midtown, Atlanta, USA

**Keywords:** hospital-based medicine, adult hospital medicine, inpatients, viral myocarditis, covid-19, post-covid myocarditis

## Abstract

While coronavirus disease 2019 (COVID-19) infection rates have declined, and mortality outcomes have improved with vaccines, targeted antiviral therapies, and improved care practices over the course of the pandemic, post-acute sequelae of SARS CoV-2 infection (PASC, also referred to as “long COVID”) has emerged as a significant concern, even among individuals who appear to have fully recovered from their initial infection. Acute COVID-19 infection is associated with myocarditis and cardiomyopathies, but the prevalence and presentation of post-infectious myocarditis are unclear. We provide a narrative review of post-COVID myocarditis, including symptoms and signs, physical exam findings, diagnosis, and treatment strategies.

Post-COVID myocarditis has a wide range of presentations, from very mild symptoms to severe ones that can include sudden cardiac death. Several studies have noted what appears to be a bimodal distribution of affected patients, with individuals under age 16 (particularly males) most affected, followed by those over age 50. The gold standard of diagnosis for myocarditis is endomyocardial biopsy and cardiac magnetic resonance imaging with a confirmed diagnosis of COVID-19. However, if these are not available, other studies such as electrocardiogram, echocardiography, and inflammatory markers can guide clinicians to diagnose post-COVID myocarditis when appropriate. Treatment is largely supportive and may include oxygen therapy, intravenous hydration, diuretics, steroids, and antivirals. Post-COVID myocarditis is rare but important to recognize as more patients present with this condition in the inpatient setting.

## Introduction and background

Since the first coronavirus disease 2019 (COVID-19) cases were diagnosed in the United States in January 2020, SARS-CoV-2 (the etiologic agent of COVID-19) has reshaped the American healthcare landscape. While infection rates have declined, and mortality outcomes have improved with vaccines, targeted antiviral therapies, and improved care practices over the course of the pandemic, post-acute sequelae of SARS CoV-2 infection (PASC, also referred to as “long COVID”) has emerged as a significant concern, even among individuals who appear to have fully recovered from their initial infection [1,2]. 

Acute COVID-19 infection is associated with myocarditis and cardiomyopathies; while the prevalence and presentation of post-infectious myocarditis are unclear, hospitalists may see many new cases over the coming years. Numerous studies have reported ongoing cardiopulmonary PASC symptoms and signs in apparently recovered patients, including those with seemingly mild infections [3,4].

In this review, we describe post-COVID myocarditis presentations, prognosis, and treatment, specifically within the hospital setting. Relatively little literature exists in this area to date. We sought to identify literature relevant to hospitalists treating adult patients for collation into a narrative review to guide them in identifying, understanding, and treating patients with post-COVID myocarditis and cardiomyopathy. 

We performed an initial literature search of PubMed and found that, depending on the search terms used, we either found thousands of potentially relevant papers and/or many non-relevant papers. Using a search strategy developed with support from our institution’s Clinical Informationists, we searched PubMed, Web of Science, and EMBASE databases for papers addressing the presentation, diagnosis, and treatment of post-COVID cardiomyopathy. We imported the results into EndNote citation management software (Clarivate Inc., Chandler, AZ, USA), eliminated duplicates, and performed a title review followed by abstract review, using inclusion and exclusion criteria (see Table 1).

**Table 1:** Literature review search terms and inclusion/exclusion criteria PASC: Post-acute sequelae of SARS CoV-2 infection

Search terms	Inclusion criteria	Exclusion criteria
“cardiomyopathies OR cardiomyopath* OR myocarditis” and “(long OR post OR 'pasc' OR 'post acute') AND ('COVID 19'/exp OR 'COVID 19' OR 'coronavirus infections'/exp OR 'coronavirus infections' OR (('coronavirus'/exp OR coronavirus) AND ('infections'/exp OR infections)) OR 'COVID'/exp OR COVID OR 'sars cov 2'/exp OR 'sars cov 2').”	Articles published in the English language	Articles that had a veterinary, pediatric, or solely geriatric focus
	Articles focused at least in part on the hospital/inpatient setting	Articles that focused exclusively on the outpatient setting
		Articles that reviewed bacterial/septic illness, vaccine/medication-related myocarditis, and all other types of non–COVID-19-related myocarditis (e.g., auto-immune [unless a new flare was brought about by the viral infection], toxic/drug related, bacterial/fungal/parasitic types)
		Articles on acute COVID cardiomyopathy
		Articles/guidance documents for following up with athletes prior to return to sports
		Articles/documents describing incidental presentation of PASC cardiac complications found in screenings of athletes
		Study protocols
		Articles examining thrombotic events
		Articles focused on subclinical disease

For papers determined to be directly salient following title review, we utilized PubMed’s Cited By and Similar Articles features and imported any potentially relevant papers into EndNote for abstract review. Using EndNote, we produced annotated bibliographies with abstracts grouped by topic area, selected the most salient, and reviewed the full texts for important points to include in the review. During drafting of the manuscript, several highly relevant records focusing on myocarditis during acute COVID-19 infection were added back to provide additional context.

## Review

Pathophysiology and observed presentations

Pathophysiology

Myocarditis is inflammation of the myocardium arising from a variety of infectious and non-infectious etiologies. Common viruses associated with viral myocarditis include adenovirus, Coxsackie B virus, cytomegalovirus, Epstein-Barr virus, parvovirus, and influenza A and B [5]. Viral infections are thought to cause myocarditis through both direct cellular injury and immune-mediated cytotoxicity via interleukin 6-mediated T-lymphocyte activation and inflammatory cytokine release [6].

Association with COVID-19 Infection

Since the start of the COVID-19 pandemic, many cases of extrapulmonary complications of COVID-19 infection have been observed, including myocarditis. In a study done by the Centers for Disease Control and Prevention (CDC), patients with COVID-19 had 15.7 times the risk for myocarditis compared to patients without COVID-19 infection [7]. A study of >150,000 US veterans found that individuals with COVID-19 are at increased risk of cardiovascular disease including myocarditis beyond 30 days post-infection, even those who were not hospitalized during active infection [8].

Cardiac involvement of COVID-19 infection is an important clinical consequence to recognize. One study reported cardiac magnetic resonance imaging (MRI)-proven myocardial inflammation in 60% of patients who had recently recovered from COVID [9]. Another study showed that 7%-20% of patients with COVID-19 had elevated cardiac biomarkers or electrocardiogram (EKG) abnormalities indicating underlying myocardial injury or ischemia [10]. A third study showed that up to 20%-30% of patients hospitalized with COVID-19 have evidence of myocardial involvement [11].

Symptoms and Manifestations

Post-COVID myocarditis can have a diverse presentation ranging from mild symptoms of fatigue, activity intolerance, chest pain, and shortness of breath to more severe presentations including cardiac arrhythmias, fulminant cardiogenic shock, or sudden cardiac death [6,12-14]. Patients can also present with more vague symptoms, including nausea, vomiting, diarrhea, myalgias, and headache. The variable presentation of myocarditis is a direct result of the extent of the myocardium affected. Myocardial inflammation can be focal or diffuse and can involve single or multiple chambers of the heart [12,13]. COVID-related myocarditis appears to typically occur after the acute phase of COVID-19 infection. Patients often have no respiratory symptoms at the time of myocarditis diagnosis [13,15].

Myocarditis can be classified as acute, chronic, or fulminant [6,12]. Acute myocarditis is generally defined as symptoms developing over ≤3 months, while chronic myocarditis is defined as symptoms developing over >3 months. Some experts further classify chronic myocarditis into chronic active myocarditis versus chronic persistent myocarditis, based on differences in histopathology. Chronic active myocarditis involves frequent clinical relapses and development of ventricular dysfunction with fibrosis noted on endomyocardial biopsy. Chronic persistent myocarditis typically has a less distinct onset of symptoms, a persistent histologic infiltrate, and no ventricular dysfunction. Fulminant myocarditis occurs when a patient presents with cardiogenic shock. These patients may experience cardiovascular compromise requiring advanced circulatory support, such as inotropes, intra-aortic balloon pumps, or extra-corporeal membrane oxygenation [12].

There have been several reports of patients with COVID-19-related myocarditis presenting with new arrhythmias, most frequently atrial fibrillation or atrial flutter. Several mechanisms have been proposed by which COVID-19 myocarditis could result in a pro-arrhythmic state. In the acute phase of infection, direct viral injury to the cardiac myocytes can result in disruption of the plasma membrane that in turn affects conduction. Sympathetic nervous system activation also increases cardiac myocyte arrhythmogenicity. After healing from the acute phase of myocarditis, some patients may develop myocardial scarring, which can predispose these patients to re-entrant tachycardias. Some COVID-19 patients can also develop chronic myocarditis, putting them at risk for myocardial scarring and re-entrant tachycardias [15]. 

Post-COVID-19 myocarditis can also present similarly to acute coronary syndrome, as reported in a case report by Noori et al. [13]. In this case, a 44-year-old male patient presented to the clinic with vague symptoms of diffuse myalgias and dry cough after having COVID-19 pneumonia one month earlier. On EKG in the clinic, he was noted to have ST-elevations in the anterolateral leads and was transferred to the emergency department with an ST elevation myocardial infarction (STEMI). He underwent cardiac catheterization that showed no significant coronary artery disease. However, his troponin was elevated to 11.67. He was admitted with a presumed diagnosis of myocarditis and had an echocardiogram showing a reduced ejection fraction of 40%. He then underwent cardiac MRI, which confirmed the diagnosis [13].

The association between COVID-19 infection and myocarditis appears to be higher for younger patients (<16 years, especially males) [16] and older patients (≥50 years) [7,17]. Patients aged 25-39 years appear to be at a lower risk. However, it has been theorized that patients in the age group of 25-39 may experience less severe disease, be less likely to seek medical care, and thus have a diagnosis of COVID-19 in the medical record, which would then in turn affect data collection. Studies also suggest that males are at higher risk of developing myocarditis in the setting of recent COVID-19 infection compared to females [7,17].

Myocardial injury during acute COVID-19 infection has been associated with increased mortality and worse outcomes [18]. This has prompted investigation into the long-term cardiovascular effects of COVID-19 infection. The World Health Organization has defined "long COVID"/PASC as the persistence of symptoms >3 after COVID-19 infection that are not explained by any other illness. In the United States, the reported incidence of PASC has ranged from 16% to 53%. PASC can involve many organ systems and produce a variety of symptoms, but cardiovascular symptoms typically reported are chest pain, palpitations, shortness of breath, and syncope [4]. Female sex, obesity, asthma, older age, and poor pre-pandemic mental health all appear to be risk factors for developing PASC. The mechanisms through which patients develop cardiac damage after acute illness with COVID are still not well understood. Some authors have theorized a chronic inflammatory response triggered by persistent viral reservoirs in the heart that lead to tissue damage and fibrosis of cardiac myocytes; this could result in impaired ventricular compliance/contractility, as well as impaired perfusion [4,19]. Another proposed mechanism is molecular mimicry causing an autoimmune attack on cardiac tissue [4].

Diversity of Symptoms at Presentation

Overall, patients with acute COVID-19 myocarditis tend to be more ill than patients who are diagnosed with post-COVID-19 myocarditis. The most common signs and symptoms are tachycardia, dyspnea, fever, cough, chest pain, post-exertional fatigue, and syncope [20,21]. Dyspnea was noted in more than three-quarters of patients: COVID-19 myocarditis causes more issues with respiratory distress than other sequelae of COVID. The number of patients who have had shock associated with COVID-19 myocarditis is high; 52% were in shock on presentation to the hospital, and the mortality rate was 14% [20]. General deconditioning was the most common symptom observed among individuals with PASC myocarditis [22].

There are two methods of cardiac damage during acute COVID-19 illness, direct and indirect. The direct method is viral infiltration of cardiomyocytes [11]. Autopsy results from COVID-19 patients have found virus within the heart tissue of more than 50% [23]. The indirect method is mediated more by hypoxia and cytokine release into the body, leading to systemic inflammation and ultimately dysregulation of the renin-angiotensin-aldosterone system [11,12] and eventually fibrosis and scarring of cardiac tissue [24]. In PASC, the mechanism is still unclear, but experts hypothesize that the damage incurred during the acute phase of viral infection could lead to post-inflammatory damage, immune system dysregulation, poor antibody response, and other complications [25]. The high cytokine release damage can cause thrombotic events, decreased oxygen supply, coronary plaque rupture, worsened cardiac reserve, increased metabolic demand, and ultimately potential progression of cardiovascular disease [26].

Diagnosis

Physical Exam Findings

Generally, myocarditis is suspected when patients with history of COVID-19 present with symptoms of chest pain/discomfort, palpitations, dyspnea, or intractable fatigue; however, these symptoms are common and non-specific [3]. In a study of 56 consecutive patients with clinically suspected post-COVID myocarditis and similar symptoms, only one patient had cardiac MRI findings confirming myocarditis and none of the patients fit the Lake Louise criteria (LLC) used to detect signs of myocardial edema, hyperemia, and fibrosis/necrosis and therefore confirm suspected myocarditis using MRI [27]. It is important to perform thorough cardiopulmonary exams to screen for irregular pulse, brady- or tachycardia, and symptoms of volume overload to detect associated arrhythmia or ventricular dysfunction leading to heart failure. Case reports of post-COVID myocarditis accompanied by other post-COVID complications such as retropharyngeal infections or stroke highlight the importance of thorough physical examinations tailored to presenting symptoms [28,29].

Imaging and Laboratory Testing

Diagnosis of post-COVID myocarditis is usually multi-modal and follows confirmation of COVID-19 infection using standard polymerase chain reaction (PCR) and seroconversion assays. The American College of Cardiology consensus statement on evaluation of cardiac involvement in active COVID-19 patients provides a framework that is also applicable to post-COVID myocarditis patients [30]. Endomyocardial biopsy and cardiac MRI criteria are considered the gold standard of diagnosis for myocarditis [31]. Initial studies performed in suspected post-COVID myocarditis patients include EKG and Holter monitoring, echocardiography, and cardiac troponin. Other inflammatory and autoimmune markers such as C-reactive protein and anti-heart antibodies have been reported to be elevated [31,32], and lactate is elevated in severe cases of cardiopulmonary collapse [33]. It is important to rule out coronary obstruction and assess ventricular function using cardiac biomarkers including enzymes and brain natriuretic peptide, and angiography when indicated [27,34]. Case reports of post-COVID myopericarditis presenting as STEMI have been published [13].

For myocarditis diagnosis, the updated 2018 LLC require a T1-based criterion (increased T1 relaxation time, extracellular volume, or late gadolinium enhancement (LGE)) and a T2-based criterion (increased T2 relaxation time, myocardial edema, increased T2 signal intensity) [35,36]. These findings are observed in post-COVID myocarditis including LGE and increased T1 relaxation time [31,37,38]. The prevalence of cardiac MR findings in patients with clinically suspected post-COVID myocarditis varies between 2% and 10% with a higher percentage showing localized edema [27,38-41]. This is notably higher than the reported incidence of post-COVID myocarditis (<1%) [42]. It is important to note that edema present and detected by cardiac MRI during COVID-19 infection can last several months after resolution of the disease [43], and evidence of cardiac involvement/limited myocardial injury without impaired blood flow is common (>50%) in recovered asymptomatic COVID-19 patients [9,44-46]. Furthermore, elevated troponin during COVID-19 was associated with MRI findings of myocardial injury in about half and evidence of prior myocarditis in about a quarter of patients at a mean of 4 months after COVID hospitalization [17,47]. Conversely, normal cardiac MRI early in the post-COVID period does not rule out later development of myocarditis [48]. Echocardiographic imaging is important and can offer clues to diagnosis using global longitudinal strain to screen for associated ventricular dysfunction [49,50], but that is not always necessarily present [31], or the presence of left ventricular thrombus associated with wall motion abnormalities [51]. Right ventricular involvement has been reported in post-COVID myocarditis and should be evaluated with either imaging modality [52].

Most Common Overlapping and Alternate Diagnoses

A small prospective study of imaging- and/or biopsy-confirmed disease in 15 patients categorized associated disease phenotypes into arrhythmic and decompensated variants [31]. Arrhythmias are variable and can include ventricular arrhythmias, supraventricular tachycardia, and atrial fibrillation without ventricular tachycardia, whereas the decompensated variant featured biventricular dysfunction in this study. The decompensated variant was uniquely associated with persistent coronavirus infection and high titers of anti-heart antibodies in this study. A case report exists of a severe presentation of heart failure symptoms coinciding with arrhythmia and delayed by several months after COVID-19 resolution [34]. Treatment required inotropic agents and a prophylactic external defibrillation vest, and full recovery was possible with guideline-directed medical therapy (GDMT), although other studies showed limited success in cases with severely reduced ejection fraction <30% [34,53]. Case reports also exist of post-COVID myocarditis associated with incomplete or complete heart block and atrial fibrillation of rapid ventricular response and ventricular dysfunction [54,55].

While some studies show a high burden of post-COVID myocarditis [17], other studies show no increase in the incidence of myocarditis requiring hospitalization after COVID infection [56]. The authors of the latter study highlight the importance of confounding factors such as other viral infections or non-infectious etiologies causing myocarditis in the post-COVID period in observational studies without appropriate control groups. Similarities between post-COVID myocarditis and other causes of idiopathic and viral myocarditis [11], as well as other systemic inflammatory syndromes that can follow viral illness (notably Kawasaki disease), have led to comparisons in mechanisms and treatment [57]. While post-COVID myocarditis is not restricted to younger patients, it is more prevalent in the younger male population. Immune activation, in particular immune complex formation and neutrophil activation, exhibits parallel processes and may offer clues to treatments that can be effective across both syndromes [58,59]. Case reports of post-COVID myopericarditis secondary to multisystem inflammatory syndrome in adults prompt a similar comparison, and GDMT and steroid treatment help with recovery [60,61].

Treatment

Treatment needs to be tailored to the variable phenotype of the disease and reflect the need to manage arrhythmia and/or decompensated ventricular function. Inflammatory markers such as auto-immune panels (anti-heart antibodies, C-reactive protein, antineutrophilic cytoplasmic antibody) point to inflammation as a possible mechanism and potential target for treatment.

Treatment for viral myocarditis is largely supportive as is the treatment for COVID-19 myocarditis. In the acute state of the disease, patients with severe disease are generally hospitalized. They may receive oxygen therapy, intravenous (IV) hydration and diuretics if they have symptoms of acute heart failure on top of myocarditis. A review by Haussner et al. found that patients with COVID-19 myocarditis received mainly IV hydration, diuretics, steroids, and antivirals (especially the latter two among patients with lung involvement) [20]. For patients with more severe disease, pressor or inotropic support was provided.

In viral myocarditis, beta-blockers may be helpful in patients with arrhythmias but can precipitate cardiogenic shock, so the clinical picture must be carefully examined [30]. Steroids are a mainstay of treatment for patients with COVID-19 and should be considered in patients with COVID-19 myocarditis; one small case series found favorable prognoses with such treatment [62]. Treatment of post-COVID myocarditis has not been widely studied, and there are no specific pharmacologic treatments for post-acute COVID syndrome myocarditis [63].

Unfortunately, there are little data on how post-COVID myocarditis may affect patients in the long term. However, we may be able to extrapolate with data we have from patients with viral myocarditis. In a study of patients with biopsy-proven inflammatory carditis, patients had a higher incidence of sudden cardiac death and shocks from their implantable cardioverter-defibrillator devices [64]. Patients were more prone to atrial and ventricular arrhythmias, and some had lower ejection fraction [65]. Post-COVID myocarditis may affect patients similarly [66].

## Conclusions

Over time, the long-term impacts of COVID-19, including myocarditis, will become clearer. Though rare at the present, post-COVID myocarditis is an important diagnosis to remember, particularly since its incidence may increase among patients in the inpatient setting over time. Post-COVID myocarditis will likely be most serious among patients who had existing cardiovascular complications. As previous cardiovascular disease is a risk factor for more severe complications of COVID-19, this will become more salient as the US population ages. Patients with post-COVID myocarditis should be followed over time to see how their disease progresses, to identify other cardiovascular complications that may present, and to elucidate optimal treatment and management. 
